# *MAT1-1-3*, a Mating Type Gene in the *Villosiclava virens*, Is Required for Fruiting Bodies and Sclerotia Formation, Asexual Development and Pathogenicity

**DOI:** 10.3389/fmicb.2020.01337

**Published:** 2020-06-25

**Authors:** Mingli Yong, Junjie Yu, Xiayan Pan, Mina Yu, Huijuan Cao, Zhongqiang Qi, Yan Du, Rongsheng Zhang, Tianqiao Song, Xiaole Yin, Zhiyi Chen, Wende Liu, Yongfeng Liu

**Affiliations:** ^1^Institute of Plant Protection, Jiangsu Academy of Agricultural Sciences, Nanjing, China; ^2^State Key Laboratory for Biology of Plant Diseases and Insect Pests, Institute of Plant Protection, Chinese Academy of Agricultural Sciences, Beijing, China

**Keywords:** mating type gene, *Villosiclava virens*, *MAT1-1-3*, sexual development, pathogenicity

## Abstract

*Villosiclava virens* is the prevalent causative pathogen of rice false smut, a destructive rice disease. Mating-type genes play a vital role in the evolution of mating systems in fungi. Some fungi have lost *MAT1-1-3*, one of the mating-type genes, during evolution, whereas others still retain *MAT1-1-3*. However, how *MAT1-1-3* regulates the sexual development of heterothallic *V. virens* remains unknown. Here, we generated the *MAT1-1-3* mutants, which exhibited defects in vegetative growth, stress response, pathogenicity, sclerotia formation and fruiting body maturation. An artificial outcrossing inoculation assay showed that the Δ*mat1-1-3* mutant was unable to produce sclerotia. Unexpectedly, the Δ*mat1-1-3* mutant could form immature fruiting bodies without mating on potato sucrose agar medium (PSA) compared with the wild-type strain, most likely by activating the truncated *MAT1-2-1* transcription to regulate the sexual development. Moreover, RNA-seq data showed that knockout of *MAT1-1-3* results in misregulation of a subset of genes involved in sexual development, MAPK signaling, cell wall integrity, autophagy, epigenetic modification, and transcriptional regulation. Collectively, this study reveals that *MAT1-1-3* is required for asexual and sexual development, and pathogenicity of *V. virens*, thereby provides new insights into the function of mating-type genes in the fungi life cycle and infection process.

## Introduction

*Villosiclava virens* (Anamorph, *Ustilaginoidea virens*) is a plant pathogen that causes rice false smut (RFS), which results in substantial rice yield losses worldwide (Brooks et al., 2010; Ladhalakshmi et al., 2012; Jecmen and Tebeest, 2015; Fan et al., 2016). *V. virens* infects rice florets to produce the RFS balls, which can generate sclerotia on their surface (Ashizawa et al., 2012; Tang et al., 2013; Hu et al., 2014; Song et al., 2016; Yong et al., 2018). Additionally, *V. virens* directly affects the rice food safety by producing mycotoxins, which are harmful to humans and animals (Li et al., 2008; Fu et al., 2017; Wang et al., 2017). In addition to the completion of genome sequencing and the available transcriptome data (Zhang et al., 2014), the recently established clustered regularly interspaced short palindromic repeats (CRISPR) mediated gene knockout system provides an effective tool to study the gene function in *V. virens* (Liang et al., 2018; Fang et al., 2019; Guo et al., 2019; Yu et al., 2019). During the pathogen’s sexual life cycle, *V. virens* can produce ascospores by sexual reproduction (Zhang et al., 2014; Yong et al., 2018), which, through invasion of rice spikelets, are considered to be one of the primary infection sources of rice false smut (RFS) (Ikegami, 1960; Wang, 1995). Genetic recombination occurs during sexual reproduction when two compatible strains mate, thereby playing a vital role in expanding *V. virens* genetic diversity (Sun et al., 2013; Wang et al., 2014). Thus, the sexual reproduction plays an important role in the prevalence of RFS and genetic diversity in *V. virens.*

Sexual reproduction is a key step for fungi to complete their life cycle (Zhang et al., 2014). In ascomycetous fungi, sexual reproduction, controlled by mating type alleles/idiomorphs (*MAT*) (Yun et al., 1999; Zheng and Wang, 2013), is divided into three major modes: heterothallic, homothallic and pseudohomothallic (or secondary homothallic) (Whittle et al., 2011; Zheng and Wang, 2013). The two opposite idiomorphs are named *MAT1-1* (or *MATA*) and *MAT1-2* (or *MATa*) (Alby et al., 2009; Whittle et al., 2011). Homothallic (self-compatible) species carry both *MAT1-1* and *MAT1-2* idiomorph genes in a single nucleus, usually closely linked or fused (Nelson, 1996; Wilken et al., 2012). In contrast, heterothallic (self-incompatible) species have alternate *MAT* idiomorph in different nuclei (Coppin et al., 1997; Yun et al., 1999). Extensive evidences showed that these mating modes could exist in different fungi belong to the same genus and play an important role in evolutionary biology. For example, the *Neurospora* species employ these three mating modes to complete the process of sexual reproduction, and multiple switches in the mating systems have occurred in the genus’ evolutionary history (Nygren et al., 2011; Whittle et al., 2011). For ascomycete *Cordyceps* sensu lato (sl), there are also reproductive switches between homothallic and heterothallic modes (Hu et al., 2013; Zheng et al., 2013). In addition, some studies supported the hypothesis that the sexual reproduction of *Aspergillus* genus may evolve from homothallic to heterothallic mode (Galagan et al., 2005; Paoletti et al., 2005), but still need more evidences to prove in the future. However, some studies supported the hypothesis that the ancestral mating system in *Neurospora* was heterothallic (Gioti et al., 2012). Compared with heterothallism, homothallism is more likely to accumulate deleterious genomic mutations (Whittle et al., 2011). To avoid self-crossing, genetic barriers have evolved sexual dimorphism to prevent selfing (Klix et al., 2010). Therefore, as one of the factors of species evolution, these studies indicate that heterothallism plays a more important role than homothallism during species evolution (Lee et al., 2010; Zheng and Wang, 2013).

*Villosiclava virens* has a heterothallic sexual reproduction system controlled by *MAT1-1* and *MAT1-2* idiomorphs (Yu J. J. et al., 2015). The *MAT1-1* idiomorph includes *MAT1-1-1*, *MAT1-1-2*, *MAT1-1-3*, and a truncated *MAT1-2-1*, and the *MAT1-2* idiomorph includes *MAT1-2-1* and *MAT1-2-8* (Yu J. J. et al., 2015). Extensive studies show that *MAT1-1* and *MAT1-2* in fungi have different functions to regulate sexual reproduction. For example, in heterothallic *N. crassa*, *matA-1* (*MAT1-1-1*) mutant is sterile but *matA-2* (*MAT1-1-2*) and *matA-3* (*MAT1-1-3*) mutants have slightly reduced fertility (Ferreira et al., 1998). In heterothallic *Podospora anserina*, *SMR2* (*MAT1-1-3*) mutant can produce asci, but the number is reduced (Zickler et al., 1995). However, in homothallic *Fusarium graminearum*, *mat1-1-2* and *mat1-1-3* mutants are fertile, *mat1-1-1* and *mat1-2-1* mutants display male- and female-specific defects, respectively (Zheng et al., 2015). In addition, in homothallic *Sordaria macrospora*, *SmtA-3* (*MAT1-1-3*) is not required for fruiting body development and also has no effect on vegetative morphology (Klix et al., 2010). In filamentous ascomycetes, *MAT1-1-1* and *MAT1-2-1* encode α-box domain proteins and high mobility group (HMG) domain transcription factors, respectively (Turgeon et al., 1993b). *MAT1-1-3* also encodes an HMG domain transcription factor (Coppin et al., 1997; Debuchy and Turgeon, 2006). In contrast to SmtA-3 (MAT1-1-3) and SMR2 (MAT1-1-3) proteins, the HMG motif is lacking in the putative SmtA-3 protein (Klix et al., 2010). Different functional requirements for *MAT1-1-3* in sexual development between homothallic *F. graminearum* and *S. macrospora* imply that some of the regulatory networks controlled by MAT proteins may not be conserved across filamentous ascomycetes (Kim et al., 2012). Thus, the function of *MAT1-1-3* homologs in sexual reproduction is variable (Yokoyama et al., 2005), and little is known about *MAT1-1-3* function in *V. virens*.

In this study, *MAT1-1-3* was characterized using Δ*mat1-1-3* mutant generated with the CRISPR/Cas9 system. We performed phylogenetic comparisons of *V. virens* with other fungi and the MAT1-1-3 proteins from different species. We have demonstrated that the transcription factor *MAT1-1-3* is required for vegetative growth, stress response, pathogenicity and sexual development in *V. virens*. The Δ*mat1-1-3* mutant could form an immature fruiting body without mating in PSA medium plate. Transcriptome analysis of Δ*mat1-1-3* mutant and wild-type strains revealed differential expression of a subset of genes. In addition, the expression level of truncated *MAT1-2-1* was up-regulated in Δ*mat1-1-3* mutant. Collectively, our study demonstrates that *MAT1-1-3* plays a vital role in sexual and asexual development in *V. virens*.

## Materials and Methods

### Strains and Growth Conditions

*Villosiclava virens* WT strains Uv1-56 (*MAT1-1* mating-type) and Uv2-51 (*MAT1-2* mating-type) and all transformants generated in this study were cultured and maintained on potato sucrose agar (PSA) plates (200 g/L potato, 20 g/L sucrose, 15 g/L agar) at 28°C in the dark. *Agrobacterium tumefaciens* strain AGL1 was incubated on LB medium (0.5% yeast extract, 1% tryptone, 1% NaCl) at 28°C. Yeast strain AH109 was incubated on YPDA medium (1% yeast extract, 2% peptone, 2% glucose, 0.003% adenine hemisulfate, 2% agar) at 30°C. All strains used are listed in Supplementary Table S1. For DNA extraction, hyphae were harvested after growth in PSA at 28°C for 7 days in the dark. Conidiation in potato sucrose broth (PSB) medium (200 g/L potato and 20 g/L sucrose) and fungal growth on PSA plates were measured using protocols from a previous study (Xie et al., 2019). Colony diameters of Uv1-56, indicated mutant and complemented strains were measured on PSA medium after 15 days at 28°C.

For conidiation, Uv1-56, mutant or complemented strains were cultured in PSB medium with shaking at 150 rpm at 28°C for 7 days. Then, the cultured mixtures were filtered, and the concentrations of conidia were measured using a hemocytometer. For the germination test, conidial suspension droplets were added to water agar plates, incubated at 28°C for 12 h and photographed with an Olympus BX-53 microscope. For stress tests, Uv1-56, mutant and complemented strains were incubated on PSA at 28°C for 15 days with different concentrations of stress agents, including 0.5 M NaCl, 0.7 M Sorbitol, 0.07% H_2_O_2_, 600 μg/ml congo red (CR), 600 μg/ml calcofluor white (CFW) and 0.03% sodium dodecyl sulfate (SDS). The inhibition rates were calculated as described previously (Xie et al., 2019). Three biological replicates were performed to assess conidiation, germination and stress tests.

### Orthology and Phylogenetic Analysis

A multisequence phylogenetic tree was constructed using the concatenated sequences of core genes identified by the default parameters of the CEGMA (v2.5) pipeline. Briefly, the CEGMA software contains the core eukaryotic genes dataset, which has 458 core genes. We searched the homologous genes in different fungal genome database using CEGMA software based on these 458 core genes. The phylogenetic tree was constructed using the homologous proteins encoded by the core homologous genes. Multiple sequence alignments of proteins were made by muscle, and then a neighbor-joining tree was built using MEGA7 with 1000 bootstrap repeats for distance estimation. The similarity of MAT1-1-3 homologs from different fungi was analyzed with MEGA7 software. The sequences of MAT1-1-3 homologous proteins in different fungi proteome database was obtained from National Center for Biotechnology Information (NCBI) with specific accession number as below: *Villosiclava virens* (*Ustilaginoidea virens*, AKE48501.1), *Metarhizium anisopliae* (BAE93596.1), *Metarhizium robertsii* (XP_007819908.2), *Ephelis japonica* (BAD72606.2), *Epichloe typhina* (BAD72610.2), *Claviceps purpurea* (BAD72602.2), *Ophiocordyceps sinensis* (AGW27558.1), *Tolypocladium inflatum* (BAE93600.1), *Pyrenopeziza brassicae* (CAA06846.1), *Fusarium graminearum* (AAG42812.1), *Fusarium fujikuroi* (AAC71053.1), *Fusarium oxysporum* (AEO15073.1), *Sordaria macrospora* (KAA8630682.1), *Neurospora crassa* (AAC37476.1), *Podospora anserina* (CAA52051.1).

### Plasmid Construction and Transformant Generation

For constructing gene replacement plasmids, the *MAT1-1-3* gene replacement vector (*pMD19-MAT1-1-3*) was obtained using previously reported methods (Li et al., 2019). Briefly, upstream and downstream flanking sequences (∼1 kb) of *MAT1-1-3* were cloned using primers *MAT1-1-3*-upF/upR and *MAT1-1-3*-doF/doR, respectively. The *hygromycin B-resistance* (*HYG*) gene fragment was amplified from the *SK1044-hyg* plasmid using primers F3/R3. The upstream, downstream and *HYG* fragments were inserted into vector T-Vector *pMD19*. To generate the *Cas9-gRNA* vector, gene gRNA spacers were designed with the gRNA designer website for best on-target scores^1^. Synthetic sgRNA oligos were annealed and inserted into *Bsm*BI-digested *pmCas9:tRp-gRNA* (Liang et al., 2018) to generate *pmCas9:tRp-gRNA*-*MAT1-1-3* constructs. The *pMD19-MAT1-1-1*, *pMD19-MAT1-1-2*, *pmCas9:tRp-gRNA*-*MAT1-1-1* and *pmCas9:tRp-gRNA*-*MAT1-1-2* constructs were generated in a similar manner. All constructs were confirmed by sequencing.

To generate knockout mutants, replacement *pMD19-MAT1-1-3* vector and *pmCas9:tRp-gRNA*-*MAT1-1-3* were co-transformed into strain Uv1-56 protoplasts using the PEG-mediated method as described previously (Li et al., 2019). *HYG* gene was used to screen for knockout transformants. *MAT1-1-3* was verified by PCR with primer pair *MAT1-1-3-*F2/R2, and the *HYG* gene was verified by PCR with primers F1/R3 and F3/R1. Primers F1/R1 were used to amplify the inserted fragment by PCR, and sequencing traces of junction regions confirmed that *MAT1-1-3* was replaced by the *HYG* gene in mutants. The *MAT1-1-1* and *MAT1-1-2* knockout mutants were also generated using the same method.

For complementation assays, the *MAT-1-1-3* gene, including its 1.5 kb promoter, was amplified with primers C-*MAT-1-1-3-*F/R. The amplified fragment was cloned into *Bam*HI and *Hin*dIII digested *pKO1-NEO-Uv* vector and was verified by sequencing analysis. The complementary vector was transformed into *A. tumefaciens* strain AGL1 and complementation transformants generated by the Agrobacterium-mediated (ATMT) were screened with geneticin selection using methods described previously (Yu M. N. et al., 2015). All primers used in this study are listed in Supplementary Table S2.

### Pathogenicity and Sclerotia Formation Assays

To detect pathogenicity, the rice susceptible cultivar Liangyoupeijiu was artificially inoculated with *V. virens* as described previously (Tang et al., 2013; Yu J. J. et al., 2015) with minor modifications. Briefly, the rice plants grown in a greenhouse were used to inoculate with *V. virens*. Uv1-56, Uv2-51, complemented and mutant strains were cultured in PSB at 28°C for 7 days with rotary shaking at 150 rpm/min. Mixtures of hyphae and conidia were created with a blender, and the conidia concentration was adjusted to 1 × 10^6^/mL with PSB. Seven days before the heading stage, about 1–2 mL of inoculum suspension was injected into 15 swollen flag leaf sheaths of rice using sterilized syringes. Inoculated rice was cultured in the greenhouse for 6 days at 25°C at 95%–100% humidity (RH) and then were placed at 28°C and about 80% RH. The rice false smut balls were counted after 30 dpi. The average diseased grain rate was calculated as previously described (Hu et al., 2014) and modified as follows: average diseased grain rate = (average of the number of false smut balls per panicle/average of the total number of rice grains per panicle) × 100%.

For sclerotia formation assays, the hyphae and conidia suspension of Uv1-56 and Uv2-51 prepared as above were mixed in a 1:1 ratio to generate the mixed inoculum suspension. The mixed inoculum suspension of Δ*mat1-1-3* and Uv2-51 (or complemented strain and Uv2-51) was also prepared using the same method. The mixed inoculum suspension was inoculated the rice swollen flag leaf sheath of rice on the seventh day before heading stage using a needle syringe. The inoculated rice plants were grown in a greenhouse at 25°C and 95%–100% RH for 6 days. Then these plants were transferred to another growth room under normal conditions (25°C-30°C, 80% RH, and 12-hr light/12-hr dark photoperiod) for 24 days of growth. Finally, these plants were further transferred to a growth incubator at a day-and-night temperature of 25°C/15°C and 70% RH for another 15 days of growth before counting the sclerotia. The sclerotium formation rate was calculated as follows: sclerotium formation rate = (average of the number of sclerotia/average of the number of false smut balls) × 100%. The sclerotia germination assay was performed according to previous methods (Yong et al., 2018). The statistical analyses were performed by Student’s t-test. All experiments were repeated three times.

### Confrontation Assay

The WT Uv1-56, Uv2-51 and mutant strains were cultured on PSA plates for 15 days. The mycelium plugs from 15-day-old PSA cultures were placed onto the right and left sides of the PSA plates, respectively. Thirty plates replicates were performed in each experimental treatment. Hyphae of Uv1-56 and Δ*mat1-1-3* were harvested at 10 and 15 days for RNA extraction. Meanwhile, the cell fusion of opposing mating type strains was observed and photographed with an Olympus BX-53 microscope. In order to observe the formation of fruiting bodies, the strain culture time was extended to 60 days and photographed with a digital camera. Three biological replicates were performed.

### qRT-PCR and RNA-seq Analysis

Gene expression was evaluated by quantitative real-time PCR with specific primers. For the confrontation assay, Uv1-56 or Δ*mat1-1-3* was crossed with Uv2-51. Samples of confrontation culture strains were harvested at 10 and 15 days. RNA was extracted with the BioTeKe RNA reagent Kit and cDNA reverse transcription was performed using the PrimeScript RT reagent Kit with gDNA Eraser (Perfect Real Time, Takara). An ABI PRISM 7000 Sequence Detection System (Applied Biosystems, United States) with SYBR^®^ Premix Ex Taq (Tli RNaseH Plus) (Takara, Japan) was used. The reactions were conducted as described previously (Fang et al., 2019). β*-tubulin* was used as the internal reference for measuring gene expression. Relative expression was determined using the 2^–ΔΔCt^ method (Livak and Schmittgen, 2001). For detecting the transcript level of *MAT1-1-3* and truncated *MAT1-2-1* in wild-type Uv1-56, mycelia were harvested after incubation in PSB at 28°C for 7 days for RNA extraction. Transcript amounts were calculated using the 2^–ΔCT^ method in ABI 7000 System Sequence Detection Software. Three biological replicates were performed and the results showed similar trends.

RNA from wild-type Uv1-56 and Δ*mat1-1-3* mutant, which were harvested after incubation on PSB at 28°C for 7 days, was extracted using Trizol^®^ Reagent. RNA-seq libraries were prepared with the ScriptSeq v2 kit (Epicentre SSV21124) following a published method (Jiao et al., 2018). Transcriptome sequencing was performed by Illumina HiSeq 2500 with the paired-end 2 × 150 bp model at the Majorbio biomedical technology Co., Ltd. (Shanghai, China). RNA-seq data from each sample were aligned to version 1 of the *Villosiclava virens* reference genome (Zhang et al., 2014) using Hisat2. Transcript assembly was performed using Cufflinks (Trapnell et al., 2012). Analysis of the difference in gene expression was performed using DEGseq. The cutoff for differential expression was transcripts per million reads (TPM) adjusted *p* < 0.05 and |log2FC| ≥ 1.

### Generation of *MAT1-1-3*-*GFP* Fusion Transformants and Subcellular Localization

To obtain the *MAT1-1-3-GFP* construct, a cDNA fragment of the *MAT1-1-3* gene was amplified from strain Uv1-56 cDNA template with primers *MAT1-1-3*-GFP-F/R and cloned into *Bam*HI and *Sma*I digested *pKD1GFP* with the ClonExpress II One Step Cloning Kit (Vazyme, China). The resulting plasmid was confirmed by sequencing analysis. The *pKD1GFP* plasmid was transformed into *A. tumefaciens* strain AGL1 using the freeze-thaw method (Weigel and Glazebrook, 2006) and was then transformed into *V virens* via ATMT transformation as described previously (Yu M. N. et al., 2015). After 7 days of *A. tumefaciens* strain and *V. virens* co-culturing, transformants were picked and observed under a fluorescence microscope. *MAT1-1-3-GFP* fusion transformants were incubated on PSB at 28°C for 7 days. The hyphae and conidia were harvested and stained with 4′, 6-diamidino-2-phenylindole (DAPI) (Sigma-Aldrich, United States) to visualize nuclei as described previously (Li et al., 2015). A rotary laser confocal microscope (Ultra View VoX, PerkinElmer, United States) was used to observe MAT1-1-3 colonization.

### 4′,6-Diamidino-2-Phenylindole (DAPI) and Calcofluor White Staining

The conidia and hyphae of indicated strains were harvested after 7 days in PSB. Staining was conducted as described previously (Li et al., 2015). To observe the septum of hyphae, the indicated strains were stained with 20 μg/ml calcofluor white (CFW), which was used to stain the cellulose and chitin in the cell wall (Roncero and Durán, 1985), for 5–15 min, and the pictures were taken by a fluorescence microscope (Nikon Eclipse 80i, Japan). The hyphae and conidia were and stained with 20 μg/mL 4′, 6-diamidino-2-phenylindole (DAPI) (Sigma-Aldrich, United States) for 1 h to visualize nucleus under the PerkinElmer UltraView VoX confocal (PerkinElmer, United States).

### Yeast Two-Hybrid Assay

Protein-protein interactions were performed by the yeast two-hybrid system (Clontech, Laboratories, Inc.). *MAT1-1-3* ORF was amplified using primers BD113-F/R, from the cDNA of Uv1-56, and then was cloned into the *Sma*I-digested *pGBKT7* vector using the ClonExpress II One Step Cloning Kit (Vazyme, Nanjing, China). The same method was used to clone *MAT1-1-1* and *MAT1-1-2* ORFs into the *Sma*I-digested *pGADT7* vector to make prey constructs. The resulting bait and prey constructs were confirmed by sequencing analysis and co-transformed into yeast strain AH109. The transformants were grown on the SD-Trp-Leu and SD-Trp-Leu-His-Ade medium plates at 30°C for 3–5 days. This experiment was repeated three times.

## Results

### Phylogenetic Analyses of *V. virens* and MAT1-1-3 Proteins With Other Fungi

**FIGURE 1:**
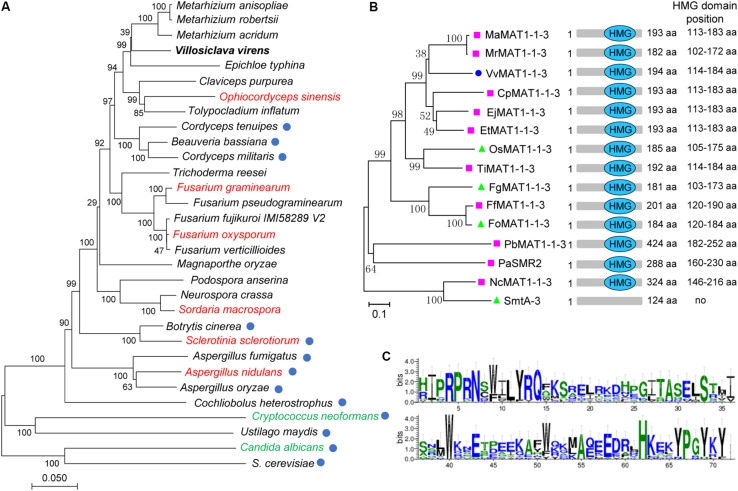
Phylogenetic analysis of *Villosiclava virens* with other fungi and VvMAT1-1-3 protein with MAT1-1-3 from various fungi. **(A)** The multisequence phylogenetic tree was constructed by MEGA7 with core eukaryotic genes identified using the CEGMA (v2.5) pipeline to show the evolutionary relationship of *V. virens* with different fungal species. Blue circles indicate species that do not have a *MAT1-1-3* gene. The red names indicate fungi that undergo homothallic sexual reproduction, the green names indicate fungi that undergo sexual reproduction that is homothallic and heterothallic. The black names indicated fungi that are heterothallic and possess a *MAT1-1-3* gene. **(B)** Phylogenetic tree of fungal *MAT1-1-3* genes and the position of the high mobility group (HMG) domain in different fungal *MAT1-1-3* genes. Pink squares indicate heterothallic fungi; green triangles indicate homothallic fungi; the blue circle indicates *V. virens*. **(C)** The weblogo depicts the multiple sequence alignment of HMG domains from the 14 protein sequences in **(B)**. The logo shows the conservation of various residues. The *y*-axis represents the bit score. A score of 4 on the *y*-axis means 100% conservation. The *x*-axis displays the amino acid position.

To explore the evolutionary relationships of *V. virens* with other fungi, we selected 31 representative members of fungi, including heterothallic, homothallic and combined species. Phylogenetic analysis established that *V. virens* is more closely related to Clavicipitaceae although received weak bootstrap support (i.e., *Metarhizium* spp. *Epichloe typhina*, *Claviceps purpurea*), followed by Ophiocordycipitaceae (i.e., *Ophiocordyceps* and *Tolypocladium*), then Cordycipitaceae (i.e., *Cordyceps spp. Beauveria bassiana*) (Figure 1A). The Clavicipitaceae, Ophiocordycipitaceae and Cordycipitaceae all belong to Hypocreales (Bushley et al., 2013). In Figure 1A, fungi indicated with red undergo homothallic sexual reproduction, fungi indicated with green undergo homothallic and heterothallic sexual reproduction, and the remaining fungi are heterothallic (Figure 1A). However, the sexual reproductive mode of Ophiocordycipitaceae switches between homothallism and heterothallism. For example, *Tolypocladium inflatum* is heterothallic, but *Ophiocordyceps sinensis* is homothallic (Hu et al., 2013). As shown by the blue circle in Figure 1A, some fungi have lost the *MAT1-1-3* gene during Hypocreales evolution. For example, *E. typhina* and *O. sinensis* have the *MAT1-1-3* gene, while *C. tenuipes*, *C*. *militaris* and *B*. *bassiana* have lost the *MAT1-1-3* gene and their sexual reproduction is heterothallic (Figure 1A). *V. virens* also has *MAT1-1-3*, suggesting that the gene plays an important role in the evolution of sexual reproduction. Phylogenetic analysis further showed that the MAT1-1-3 protein from *V. virens* is clustered with those from *Metarhizium* spp. (Figure 1B). In addition, all of the sequences from 15 diverse fungi were found to contain the high mobility group (HMG) domain, except SmtA-3 (Figure 1B). Thus, we further compared the HMG domain sequences of 14 fungi species using WebLogo. Multiple sequence alignment of these proteins showed that the HMG domain is conserved among different fungi (Figure 1C). Collectively, the results suggest that MAT1-1-3 is a conserved protein among different fungi.

### *MAT1-1-3* Affects *V. virens* Mycelial Growth, Conidial Morphology and Production

To investigate the function of *V. virens MAT1-1-3*, we generated Δ*mat1-1-3* mutants in strain Uv1-56 (wild type) by using the CRISPR/Cas9 system (Liang et al., 2018; Li et al., 2019) to replace *MAT1-1-3* with an *HYG* gene (*hygromycin B-resistance*) (Supplementary Figure S1A). Genome DNA PCR and sequencing analysis of three Δ*mat1-1-3* mutants (#8, #16 and #20) confirmed that *MAT1-1-3* was successfully replaced (Supplementary Figures S1B,C). These mutants had similar phenotypes, so mutant #16 was selected for additional study. A complementation assay was carried out with Δ*mat1-1-3#16* to generate the CΔ*mat1-1-3#16* complemented strain (Supplementary Figures S1B,C). The *MAT1-1-3* is located at the *MAT1-1* idiomorph of *V. virens*, which also contains two other mating type genes *MAT1-1-1* and *MAT1-1-2*. Thus, to assess whether *MAT1-1-1* and *MAT1-1-2* have the same phenotype with *MAT1-1-3*, we also generated Δ*mat1-1-1* and Δ*mat1-1-2* mutants using the same method.

**FIGURE 2:**
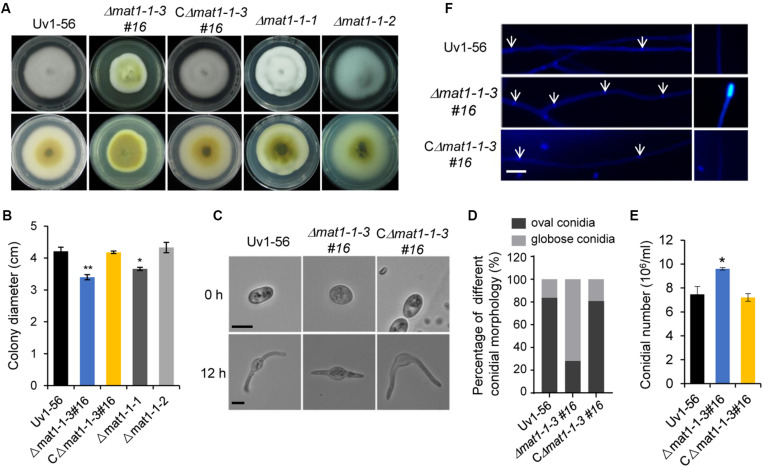
*MAT1-1-3* affects mycelial growth, conidial morphology and production. **(A)** Colony morphology of wild-type (Uv1-56), Δ*mat1-1-3#16* deletion mutant, complemented strain CΔ*mat1-1-3#16*, Δ*mat1-1-1* and Δ*mat1-1-2* deletion mutants on PSA medium after 15 days of incubation at 28°C. **(B)** Quantified growth rate of wild type, mutant and complemented strain in **(A)**. Colony diameter was statistically analyzed, and error bars represent SD. The asterisks indicate significant differences (one-way ANOVA, *p < 0.05, **p < 0.01). **(C)** The conidial morphology and germination of wild-type, mutant and complemented strains were photographed after culturing on water agar plates for 0 h and 12 h. Scale bars, 5 μm. **(D)** Statistical analysis of the percentage of different conidial morphology in wild-type, mutant and complemented strains. **(E)** Statistical analysis of conidia production on PSB medium after 7 days of culturing with 150 rpm shaking at 28°C. Data are shown as mean ± SD from three independent replicates. Asterisks indicate significant differences (one-way ANOVA, *p < 0.05). **(F)** Hyphae of wild-type, mutant and complemented strains were stained with calcofluor white, which detects chitin and cellulose in the cell wall, and fluorescence is shown in blue color. The hyphal septum of the Δ*mat1-1-3* mutant was shorter than that of wild type and complemented strains. Deletion of MAT1-1-3 altered the distribution of chitin in the cell wall. Arrows indicate the hyphal septa. Scale bars, 10 μm.

The Δ*mat1-1-3* and Δ*mat1-1-1* mutants, but not Δ*mat1-1-2* mutant, exhibited a reduction in mycelial growth rate compared with those of the WT Uv1-56 and complemented strain (CΔ*mat1-1-3#16*) (Figures 2A,B). Moreover, the colony of Δ*mat1-1-3* mutant exhibited deeper pigmentation on PSA medium plate compared with those of the WT Uv1-56, Δ*mat1-1-1*, Δ*mat1-1-2* mutants and complemented strain (Figures 2A,B). Based on the difference in colony morphology between Δ*mat1-1-3* mutant and the other two mutants (Δ*mat1-1-1* and Δ*mat1-1-2*), we only focused on studying the function of *MAT1-1-3* in this study. The Δ*mat1-1-3* mutant produced more globose conidia compared with WT and complemented strain, which produced more oval conidia (Figures 2C–E). The conidia produced by Δ*mat1-1-3* mutant geminated slower than that of produced by WT and complemented strain (Figure 2C). CFW staining showed that the hyphal septa of the Δ*mat1-1-3* mutant increased and chitin accumulated on the tip of mycelium in the Δ*mat1-1-3* mutant compared with WT (Figure 2F), indicating that *MAT1-1-3* involved in regulating cell wall chitin and cellulose synthesis. Mycelial growth and hyphal septa defects of the mutant were rescued in the complemented strain CΔ*mat1-1-3#16* (Figure 2). Together, these results indicated that *MAT1-1-3* was required for conidial morphogenesis, conidia production, germination and cell wall integrity.

### *MAT1-1-3* Regulates *V. virens* in Response to Different Abiotic Stresses

**FIGURE 3:**
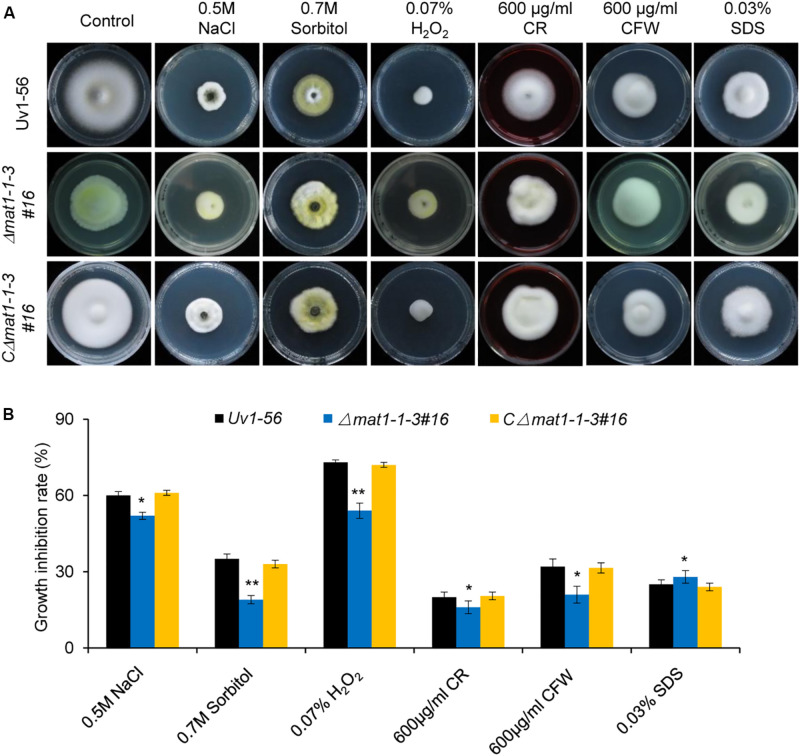
*MAT1-1-3* is involved in regulating pathogen stress responses. **(A)** Mycelial radial growth of the indicated wild-type, Δ*mat1-1-3#16* and complemented CΔ*mat1-1-3#16* strains on PSA medium supplemented with salt stress agent (0.5 M NaCl), osmotic stress agent (0.7 M Sorbitol), oxidative-stress agent (0.07% H_2_O_2_), and cell wall disturbing agents Congo Red (CR, 600 μg/mL), calcofluor white (CFW, 600 μg/mL) and sodium dodecyl sulfate (0.03% SDS). Photographs were taken after 15 days of incubation at 28°C. **(B)** Statistical analysis of the indicated strains growth inhibition rate under different stress conditions. Colony diameters of the indicated strains were measured. Data are shown as mean ± SD of three independent replicates. Asterisks indicate significant differences (one-way ANOVA, *p < 0.05, **p < 0.01).

To test whether knockout of *MAT1-1-3* affects *V. virens* responses to the osmotic, oxidative stress and cell wall integrity. The WT, mutant and complemented strains were cultured on PSA medium containing different stress agents. The growth inhibition rate of mycelial growth was calculated after 15 days of culture at 28°C. Our results demonstrated that the growth inhibition rate of mycelial growth of mutant displays significant difference, the Δ*mat1-1-3* mutant exhibited increases in the tolerance to NaCl, Sorbitol, H_2_O_2_, CR and CFW, but more sensitive to SDS compared with WT and complemented strain CΔ*mat1-1-3#16* (Figure 3). Taken together, these results indicated that *MAT1-1-3* is required for regulating the *V. virens* responses to osmotic stress and oxidation stress as well as cell wall integrity.

### *MAT1-1-3* Is Required for the Pathogenicity of *V. virens*

**FIGURE 4:**
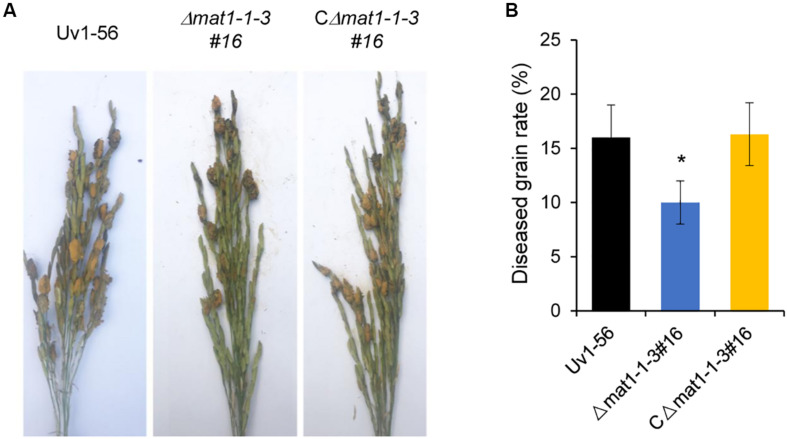
*MAT1-1*-*3* is required for *Villosiclava virens* full pathogenicity in rice. **(A)** Rice spikelets were infected with an inoculum of wild-type Uv1-56, Δ*mat1-1-3#16* mutant and complemented strains. Pictures were taken 30 days post-inoculation. **(B)** Statistical analysis of the average diseased grains rate in the infected spikelets. False smut balls were counted from each single rice panicle as described in materials and methods. Data represent means ± SD from three independent experiments. Asterisks indicate significant differences (one-way ANOVA, *p < 0.05).

To further evaluate the role of *MAT1-1*-*3* in fungal pathogenicity, suspensions of shattered hyphae and conidia from various strains were individually inoculated into booting stage rice panicles. At 30 days post-inoculation (dpi), the false smut balls produced on rice spikelets were counted to evaluate *V. virens* pathogenicity. Rice panicles infected by the Δ*mat1-1-3* mutant produced significantly fewer false smut balls than those infected by WT and complemented CΔ*mat1-1-3#16* strains (Figure 4). This result suggested that *MAT1-1-3* was required for the pathogenicity of *V. virens.*

### *MAT1-1-3* Is Required for Sclerotia Formation

**FIGURE 5:**
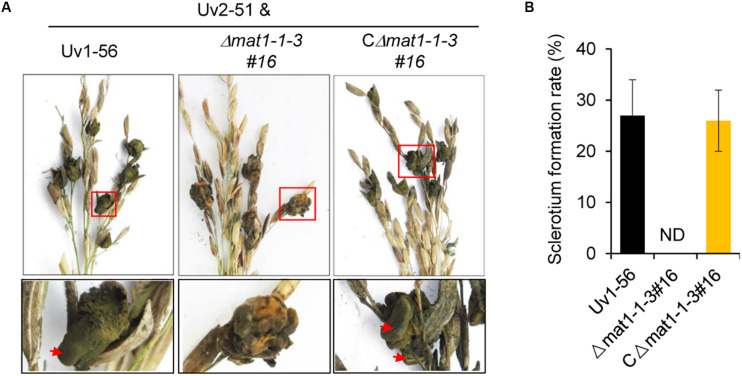
*MAT1-1-3* is indispensable for *Villosiclava virens* sclerotia formation and sexual development. **(A)** In contrast with Uv1-56 and complemented strains, the Δ*mat1-1-3#16* mutant was unable to produce sclerotia. Pictures were photographed 45 days post-inoculation (dpi). The enlarged regions marked with squares were shown at the bottom. **(B)** Statistical analysis of sclerotium formation rates of wild-type, mutant or complemented strains crossed with Uv2-51 at 45 dpi. Data are shown as means ± SD of three independent replicates. ND, not detection.

We next investigated whether *MAT1-1-3* regulated sclerotia formation of *V. virens*. On the seventh day before heading stage, the swollen flag leaf sheath of rice was inoculated with the mixed suspension of conidia and hyphae of Uv1-56 & Uv2-51, Δ*mat1-1-3* & Uv2-51, and CΔ*mat1-1-3#16* & Uv2-51. As shown in Figure 5, Δ*mat1-1-3* mutant could not produce the sclerotia, whereas the wild-type Uv1-56 could produce the sclerotia on the surface of false smut balls, which were marked by the red arrows. Apparently, the complemented strain CΔ*mat1-1-3#16* restored the sclerotia production to the WT level (Figure 5). Together, the results suggested that *MAT1-1-3* was required for sclerotia formation and sexual development of *V. virens*.

### MAT1-1-3 Is a Negative Regulator of the Immature Fruiting Body in *V. virens*

**FIGURE 6:**
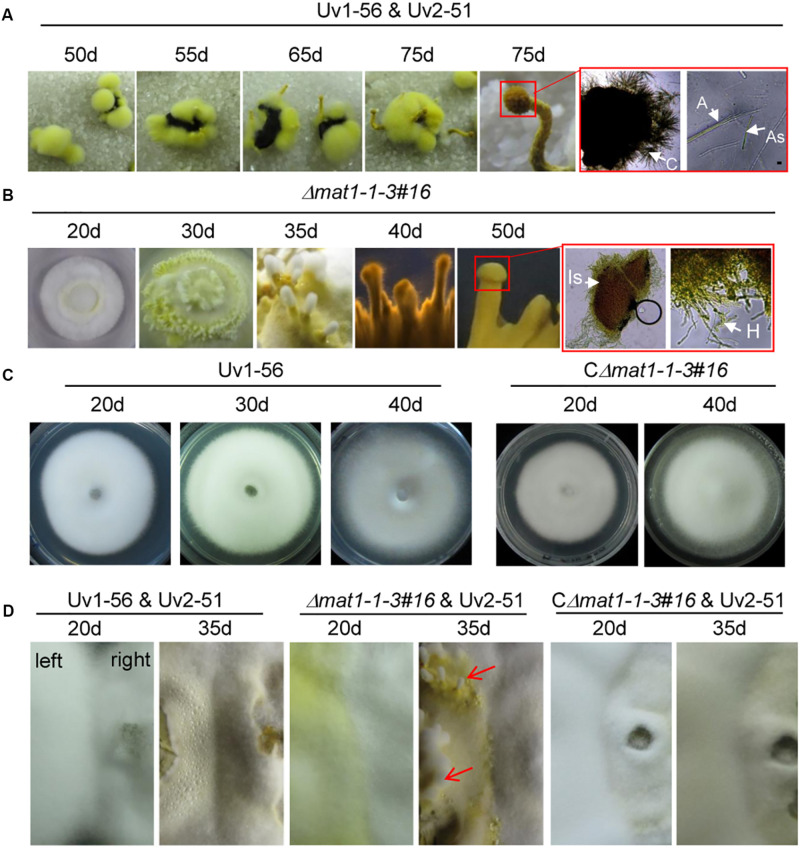
*MAT1-1-3* negatively regulates the formation of fruiting bodies in *Villosiclava virens.* **(A)** Inoculum of wild-type Uv1-56 mixed with Uv2-51 infected the rice panicle to form fertile sclerotia. Wild-type sclerotia germination formed different sexual structures at different periods. The magnified red square region indicates that mature fruiting bodies could form a cavity and ascospores. **(B)** Sexual structures for the Δ*mat1-1-3* mutant formed after different periods on the PSA medium. The magnified red square region shows that the fruiting body was immature, no ascospores were produced and only hypha formed. **(C)** Wild-type Uv1-56 and complemented CΔ*mat1-1-3#16* strains could not form the sexual structure on PSA plates. **(D)** For the confrontation assay, wild-type Uv1-56, complemented strain or mutant Δ*mat1-1-3* were confronted with wild-type Uv2-51 and cultured at 28°C for 20 days and 35 days on PSA medium. The colony growing on the right in the plate represents the Uv2-51 strain. The Δ*mat1-1-3* mutant, but not WT Uv1-56 and complemented strains, formed sexual structures at 35 days after confronting culture with Uv2-51. Is, Immature stroma; H, hypha; C, cavity; A, asci; As, ascospores.

The fertile sclerotia play an important role in the sexual cycle and development of *V. virens*, but current research shows that it is only produced in rice spikelets infected by two opposite mating-type strains in the field. The sclerotia can germinate and form different sexual structures, such as primordia, fruiting body, asci and ascospores. The germination of sclerotia collected from the field was observed in the laboratory. Germination assays showed that the sclerotia began to germinate at 50 days, primordia emerged at 55 to 65 days, and mature fruiting bodies, filled with asci containing ascospores, appeared at 75 days (Figure 6A). Although Δ*mat1-1-3* mutant could not produce sclerotia after outcrossing with Uv2-51 in the field (Figure 5), the Δ*mat1-1-3* mutant alone formed the fruiting body primordium and germinated on the PSA medium (Figure 6B). The mycelium of Δ*mat1-1-3* mutant began to grow densely at 20 days and a large number of hyphae differentiated into filaments at 30 days (Figure 6B). Then the primordia appeared at 35 days (Figure 6B). At 40 days, the primordia of fruiting body appeared (Figure 6B). However, the fruiting body could not reach maturity and the immature stromata had no ascospores (marked with red squares), even though extended the growth time in PSA medium plate (Figure 6B), indicating that the Δ*mat1-1-3* mutant alone can form similar sexual structures. Conversely, wild-type Uv1-56 and CΔ*mat1-1-3#16* could not form fruiting body primordia and the colonies began to dry and shrink after 40 days on PSA medium (Figure 6C). After crossing with Uv2-51, only the Δ*mat1-1-3* mutant could form the primordia at 35 days compared with WT and CΔ*mat1-1-3#16* (Figure 6D). Taken together, these results indicate that *MAT1-1-3* negatively regulates the formation and development of immature fruiting bodies during the sexual development of *V. virens*.

### Deletion of *MAT1-1-3* Affects Expression of Pheromone Precursor and Pheromone Receptor Genes and Mating Hyphae Growth

**FIGURE 7:**
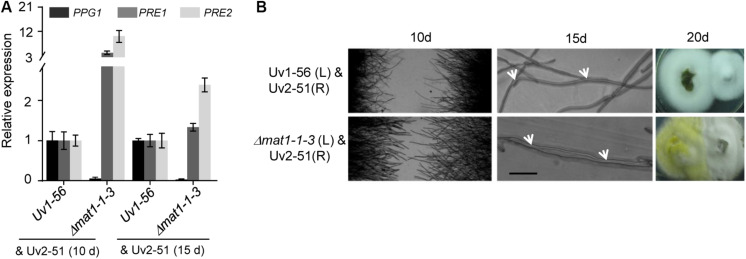
*MAT1-1-3* affects the expression of pheromone and pheromone receptor genes. **(A)** qRT-PCR analysis of *PPG1*, *PRE1* and *PRE2* in wild-type Uv1-56 and Δ*mat1-1-3* mutant. The wild-type and mutant strains were confronted with the *MAT1-2* type strain Uv2-51 after 10 days or 15 days on PSA medium. **(B)** Mating hyphae formation assay with wild-type and mutant strains confronted with Uv2-51. After 10 days, when Δ*mat1-1-3* crossed with Uv2-51, both strains formed mating hyphae that were longer than those of control Uv1-56 crossed with Uv2-51. After 15 days, hyphal fusions, indicated by arrows, appeared between Uv1-56 and Δ*mat1-1-3* mutant crossed with Uv2-51. The Δ*mat1-1-3* mutant fused earlier than wild-type with the Uv2-51 strain at 20 days. Scale bars, 10 μm.

The expression level of pheromone precursor and pheromone receptor genes are strictly regulated during sexual reproduction. Yu et al. (2016) identified several genes homologous to pheromone precursor (*PPG1*, a homolog of *ccg-4* in *N. crassa*) and pheromone receptors (*PRE1* and *PRE2*, homolog of *pre-1* and *pre-2* in *N. crassa*, respectively) in *V. virens* (Yu et al., 2016). Given that *MAT1-1-3* regulated the sexual development of *V. virens*, we investigated the expression of pheromone precursor and pheromone receptors genes after confronting mutant or WT strains with the *MAT1-2* strain Uv2-51 for 10 days or 15 days. Compared with the WT, qRT-PCR results showed decreased *PPG1* expression and increased *PRE1* and *PRE2* expression in the Δ*mat1-1-3* mutant at 10 days and 15 days, and the expression level of *PRE1* and *PRE2* at 10 days was higher than at 15 days (Figure 7A), suggesting that *MAT1-1-3* can regulate transcription of these genes. It has been known that pheromone-related genes also regulate the cell fusion of opposing mating type strains before mating (Jones and Bennett, 2011). To test if the cell fusion was affected in the Δ*mat1-1-3* mutant, we employed a confrontation assay (Snetselaar et al., 1996). In the control, mating hyphae formed normally when Uv1-56 was confronted with Uv2-51 at 10 days (Figure 7B). However, when the Δ*mat1-1-3* mutant was confronted with Uv2-51, both strains had longer mating hyphae than those in the control, and the hyphae of Δ*mat1-1-3* mutant were more frequently fused with Uv2-51 than with WT strain at 15 days (Figure 7B). At 20 days, the Δ*mat1-1-3* mutant colony fused better with Uv2-51 than with the wild-type strain (Figure 7B). Taken together, these results demonstrate that *MAT1-1-3* is likely to regulate the expression of *PPG1*, *PRE1* and *PRE2* to affect mating hyphae growth and hyphae fusion.

### Subcellular Localization and the Interacting Protein of MAT1-1-3

**FIGURE 8:**
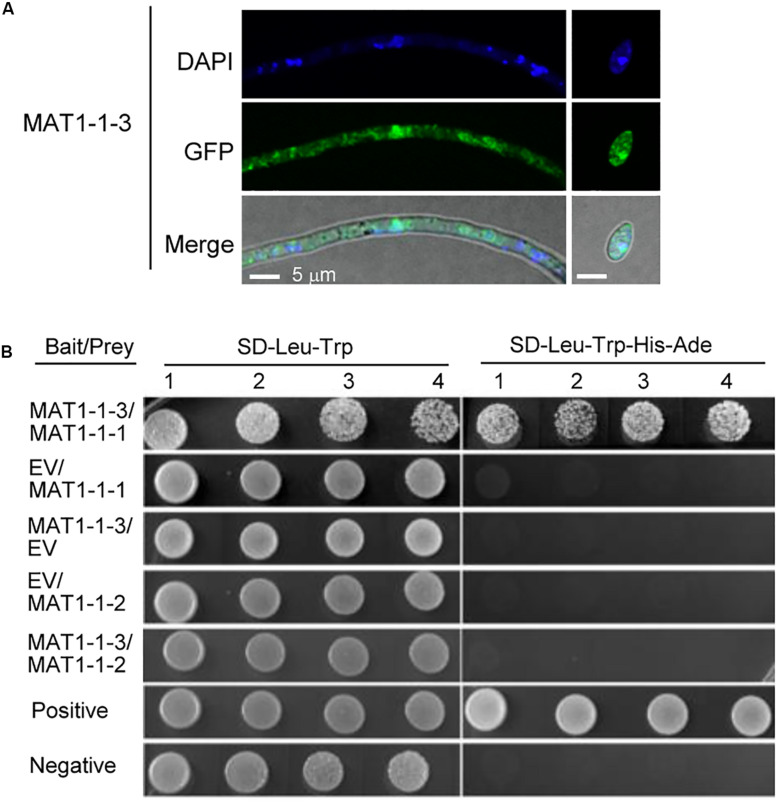
Subcellular localization and the interacting protein of MAT1-1-3 in *Villosiclava virens*. **(A)** The subcellular localization of MAT1-1-3-GFP in the hyphae and conidia of *V. virens*. The hyphae and conidia were stained with DAPI before the signal was observed with a confocal microscope. Scale bars, 5 μm. **(B)** The yeast two-hybrid (Y2H) assay was used to examine interactions between MAT1-1-1, MAT1-1-2 and MAT1-1-3. EV, empty vector; SD/-Leu-Trp-His-Ade, synthetic dropout (SD) media lacking leucine, tryptophan, histidine and Adenine; SD/-Leu-Trp, SD media lacking leucine and tryptophan. Numbers indicate repeats using different single colonies.

To better understand the biological function of *MAT1-1-3*, we examined the localization of MAT1-1-3 fused with green fluorescence protein (GFP) by a confocal microscope. The transformants expressing MAT1-1-3-GFP were generated in the background of WT Uv1-56. We observed that MAT1-1-3-GFP signal accumulated in the nucleus and cytoplasm of the conidia and hyphae (Figure 8A). The nuclear signal was confirmed by nuclear dye DAPI. The proteins encoded by mating-type genes can form a heterodimer, which is involved in morphological changes and spore development in fungi (Kämper et al., 1995; Lee et al., 2010; Kües et al., 2011). To investigate the relationship between the MAT1-1-3 and other mating-type proteins in *V. virens*, we performed a yeast two-hybrid (Y2H) assay to examine the interaction. Y2H assay showed that MAT1-1-3 interacted with MAT1-1-1, but could not interact with MAT1-1-2 (Figure 8B), indicating that MAT1-1-3 and MAT1-1-1 may form a complex to regulate sexual reproduction of *V. virens*.

### *MAT1-1-3* Deletion Affects the Transcription of a Subset of Genes

**FIGURE 9:**
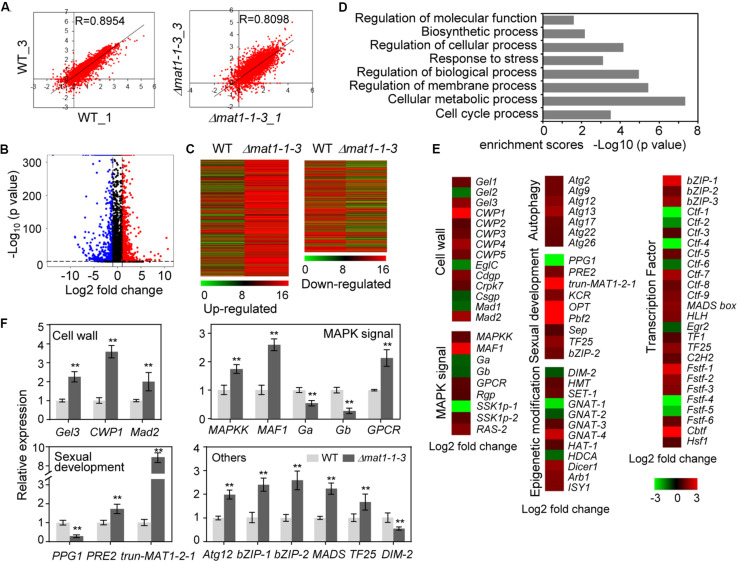
*MAT1-1-3* regulates the expression of a subset of genes. **(A)** Scatter plots showing correlation of whole-genome expression measured as transcripts per million reads (TPM) between two WT samples (WT_1 versus WT_3, left) or two Δ*mat1-1-3* mutant samples (Δ*mat1-1-3_1* versus Δ*mat1-1-3_3*, right). Gene expression levels were measured for mycelium grown in PSB for 7 days. The x-axis indicates gene expression in WT_1 or Δ*mat1-1-3_1*, and the *y*-axis indicates gene expression in WT_3 or Δ*mat1-1-3_3* mutant. **(B)** Volcano map of differently expressed genes between WT and Δ*mat1-1-3* mutant. The *x*-axis indicates relative gene expression (log2 fold change between WT and Δ*mat1-1-3* mutant), and the *y*-axis indicates the mean of normalized counts [–log_10_ (*p*-value)]. Genes with significantly up-regulated expression (*p* < 0.05 and log_2_ fold change ≥ 1) are shown in red. Genes with significantly down-regulated expression (*p* < 0.05 and log_2_ fold change ≤ 1) are shown in blue. **(C)** Heatmaps of genes up-regulated and down-regulated in the Δ*mat1-1-3* mutant compared with WT. The original means of reading counts were adjusted by log_2_ transformation using MeV4.0. **(D)** Gene ontology (GO) enrichment analysis of significantly differentially expressed genes in Δ*mat1-1-3* compared with WT. The *x*-axis indicates enrichment scores [–log10 (*p*-value)] (the *P*-value indicates the possibility of significant enrichment) for each GO item on the *y*-axis. The fold enrichment was calculated based on the frequency of genes annotated to the term compared to their frequency in all transcripts detected. **(E)** Differentially expressed genes in the Δ*mat1-1-3* mutant might indicate involvement in pathogenesis and sexual development. Regulated genes include genes involved in cell wall integrity, MAPK signaling, sexual development, autophagy, and epigenetic modification. Some genes encoding transcription factors were also regulated by *MAT1-1-3*. The full names of abbreviated genes are shown in Supplementary Table S5. **(F)** qRT-PCR analysis of *MAT1-1-3* regulated genes. Relative expression levels were normalized with β*-tubulin* as the internal standard and presented as means ± SD from three biological replicates. Asterisks indicate significant differences (Student’s *t*-test, *p < 0.05, **p < 0.01).

To further study *MAT1-1-3* regulation mechanisms in *V. virens*, we performed transcriptome sequencing (RNA-seq) analysis of the Δ*mat1-1-3* mutant. The transcripts of *MAT1-1-3* could be detected when WT cultured in potato sucrose broth (PSB) medium (Supplementary Figure S2A). Thus, the samples for RNA-seq were extracted from mycelia of Δ*mat1-1-3* mutant and WT cultured in PSB for 7 days. We prepared three biological replicates samples for Δ*mat1-1-3* (Δ*mat1-1-3_1/2/3*) mutant and WT (WT_1/2/3), respectively, though one sample from Δ*mat1-1-3* and another sample from WT were contaminated and discarded. The correlation coefficients (0.8954 and 0.8098) for the expression profiles of all transcripts between two wild-type (WT-1 and WT-3) or two mutant (Δ*mat1-1-3_1* and Δ*mat1-1-3_3*) samples, respectively (Figure 9A), suggested that the RNA-seq data were credible. Analysis of the differentially expressed genes (DEG) revealed that the *MAT1-1-3* knockout affected the transcription of a subset of genes (Supplementary Table S3). In comparison with WT, 1001 genes showed increased expression and 651 genes showed decreased expression in the Δ*mat1-1-3* mutant (Figures 9B,C and Supplementary Table S4). Enrichment analysis of Gene Ontology (GO) categories indicated that all significantly differentially expressed genes involved in regulation of molecular function, biosynthetic process, response to stress, regulation of cellular process, cellular metabolic and cell cycle process were significantly enriched (Figure 9D).

Extensive evidence has shown that genes involved in fungal cell wall integrity, MAPK signaling, sexual development, autophagy process, transcription factors and epigenetic modification are involved in fungal pathogenicity, growth and development. We further analyzed 1652 up- and down-regulated genes and found that a subset of genes involved in these processes was misregulated in the Δ*mat1-1-3* mutant (Figure 9E and Supplementary Table S5). The cell wall is essential for growth and participates in morphogenetic and differentiation processes in fungi (de Medina-Redondo et al., 2008). qRT-PCR results confirmed increased expression in Δ*mat1-1-3* mutant of genes encoding beta-1,3-glucanosyltransferase (*Gel3*), cell wall protein (CWP1) and adhesin protein Mad2 (*Mad2*) (Figure 9F). Several genes involved in the MAPK signaling pathway, which is essential for mating (Herskowitz, 1995; Banuett, 1998), including encoding the mitogen-activated protein kinase (*MAF1*), mitogen-activated protein kinase kinase (*MAPKK*) and G-protein coupled receptor (*GPCR*) increased expression in Δ*mat1-1-3* mutant; however, the expression was reduced for two genes encoding G protein alpha and beta subunit (*Ga* and *Gb*) (Figure 9F and Supplementary Table S5). Additionally, in the mutant, the expression levels of several genes involved in sexual development increased, like *PRE2*, or decreased, like *PPG1* (Figure 9F), which is consistent with our previous results (Figure 7A). Studies have reported that genes encoding transcription factors (*bZIP-1*, *bZIP-2*, *MADS-box* and *TF25*) involved in fruiting body development (Zheng and Wang, 2013; Yu et al., 2016), with significantly increased expression in the Δ*mat1-1-3* mutant (Figures 9E,F). In addition, all seven autophagy-related genes were up-regulated in the mutant, and several epigenetic modification related genes, such as DNA methyltransferase (*DIM-2*), histone methyltransferase (*HMT*) and GNAT family acetyltransferase, were misregulated in the mutant (Figures 9E,F and Supplementary Table S5). Taken together, these results suggest that *MAT1-1-3* regulates the expression of a subset of genes to control related functions in *V. virens*.

Our previous study showed that the truncated *MAT1-2-1* down-stream of the *MAT1-1-3* is part of the *MAT1-2-1* located on *MAT1-2* idiomorph (Supplementary Figure S2B) (Yu J. J. et al., 2015). The length of truncated *MAT1-2-1* is 273 bp without ATG, which shares 87% identities with the 273 bp at 3′ end of *MAT1-2-1* in the nucleotide sequence (Supplementary Figure S2C). The encoding protein with an HMG domain was predicted according to the 3′ end of MAT1-2-1 sequence, which shares 77% identities with the corresponding sequences in MAT1-2-1 (Supplementary Figure S2). RNA-seq data showed that the expression of truncated *MAT1-2-1* gene increased about 9-fold in the Δ*mat1-1-3* mutant, which was confirmed by qRT-PCR (Figures 9E,F). However, fewer transcripts of truncated *MAT1-2-1* are detected in WT compared with *MAT1-1-3* (Supplementary Figure S2A). The results suggest that *MAT1-1-3* negatively regulates the expression level of truncated *MAT1-2-1*.

## Discussion

Sexual cycle plays a crucial role in *V. virens* overwintering and genetic diversity (Sun et al., 2013; Wang et al., 2014; Deng et al., 2015; Yong et al., 2018). The sclerotia produced during the sexual cycle can survive in winter and produce a large number of ascospores in the coming year, which are considered as the primary infection source (Yong et al., 2018). Genetic recombination occurs during sexual reproduction and expands *V. virens* genetic diversity (Sun et al., 2013; Wang et al., 2014). Mating type genes play crucial roles in sexual reproduction (Zheng and Wang, 2013). Here, to better understand the genetic basis of sexual reproduction in *V. virens*, we characterized a mating type gene *MAT1-1-3* in *V. virens*. We found that MAT1-1-3 is a negative regulator of the immature fruiting body in *V. virens*, meanwhile, it is required for the asexual development and pathogenicity.

### The Evolution of *V. virens* and MAT1-1-3 Proteins

The study of evolutionary relationships contributes to better understand the fungal origin and the relationships between different fungi. Previously, Zhang et al. (2014) systematically analyzed the evolutionary relationship of *V. virens* with other eleven fungi (ten Ascomycota and one Basidiomycota outgroup), including *Metarhizium* spp. via comparative genomic analyses and proteome comparisons. This study showed that *V. virens* is more closely to *Metarhizium* spp. than to other species including the plant pathogen *Claviceps purpurea* and the insect pathogenic fungus *Cordyceps militaris*. Our phylogenetic analysis data also showed that *V. virens* and *Metarhizium* spp. are in the same cluster, implying that the evolutionary relationship is close. The sexual reproduction mode of *Metarhizium* spp. is heterothallic (Zheng et al., 2011), which is consistent with *V. virens.* In addition, phylogenetic analysis data also showed that MAT1-1-3 proteins of *V. virens* and *Metarhizium* spp. are also in the same cluster. Among 31 fungi that we analyzed, some fungi (*C. tenuipes, C. militaris* and *B. bassiana*) have lost the *MAT1-1-3* gene, however, most fungi still have it. Several researches showed that *MAT1-1-3* may have been lost during the shift from plant to insect hosts, and could be a good indicator of Clavicipitaceae evolution (Yokoyama et al., 2006; Zheng et al., 2013). Our results showed that *MAT1-1-3* of *V. virens* is required for sclerotia formation and sexual development.

### The Relationship Between Truncated Mating Type Genes and Mating Type Genes

Increasing studies show that unequal recombination/crossover and transposable elements (TE)-mediated translocations play an important role in the evolution of mating loci (Gioti et al., 2012; Dyer et al., 2016). In *Grosmannia clavigera*, the *MAT1-2* idiomorph contained a truncated *MAT1-1-1* located up-stream of the *MAT1-2-1* gene, which is homologous to the *MAT1-1-1* gene in the *MAT1-1* idiomorph (Tsui et al., 2013). Moreover, this truncated *MAT1-1-1* is present in multiple *G. clavigera* isolates. And they found that the unequal recombination/crossover event likely accounted for the truncated *MAT1-1-1*. Because the unequal recombination/crossover events have occurred in many fungal *MAT* idiomorphs. For example, the truncated *MAT1-1-1* gene was found in the *MAT1-2* idiomorph of *Hypocrea jecorina* (Seidl et al., 2009) and also was identified in the *MAT1-2* idiomorph of at least five *Phialocephala* species (Zaffarano et al., 2010). In contrast, a partial *MAT1-2-1* sequence (360bp) located in the *MAT1-1* idiomorph of *Aspergillus fumigatus* (Paoletti et al., 2005). Our study here also showed that a truncated *MAT1-2-1* was found in *MAT1-1* idiomorph of *V. virens*. The length of truncated *MAT1-2-1* is 273 bp without start codon, but contains stop codon, which shares 87% identities with the 273 bp at 3′ end of *MAT1-2-1* in the nucleotide sequence. The encoding protein with an HMG domain was predicted according to the 3′ end of MAT1-2-1 sequence. The encoding protein shares 77% identities with the corresponding amino acid sequences in MAT1-2-1. Thus, we also think that the truncated *MAT1-2-1* organization is due to unequal recombination/crossover events during sexual reproduction.

Several studies showed that recombination/crossover event caused the truncated genes (*MAT1-1-1* or *MAT1-2-1*) to be non-functional or inactive in mating, thereby being called pseudogenes. For example, the truncated *MAT1-1-1* gene in *Ophiostoma montium* is highly eroded and possible is a pseudogene (Tsui et al., 2013). Similarly, the truncated *MAT1-1-1* gene in *Cordyceps takamontana* (known as *Isaria tenuipes*) was a pseudogene due to accumulated mutations and stop codons (Yokoyama et al., 2003). Our previous study showed that the truncated *MAT1-2-1* of *V. virens* was also named a pseudogene due to no start codon and very low detectable transcripts. However, the transcripts of truncated *MAT1-2-1* increased about 9-fold after knockout *MAT1-1-3*, indicating that it may have function, thereby being named truncated *MAT1-2-1* here. These data also suggest that *MAT1-1-3* suppresses the expression of truncated *MAT1-2-1* to regulate the mating of *V. virens*. Consistently, the transgenic strains, carrying both mating type genes, are unable to produce progeny in isolates of *N. crassa* (Glass et al., 1990), *Podospora anserina* (Coppin and Debuchy, 2000) and *Cochliobolus heterostrophus* (Turgeon et al., 1993a). These results, including our data, suggest that the truncated genes may interfere or compete with the signal from the “resident/original” HMG domain at the same locus. It is also possible that the truncated genes have evolved new functions through adaptive evolution (Tsui et al., 2013). Therefore, it will be important for future studies to investigate whether the truncated genes have functions and the mechanisms of how it regulates sexual reproduction.

### Function of *MAT1-1-3* in Asexual Development, Sexual Cycle and Pathogenicity

In heterothallic *N. crassa*, the *matA-3* (*MAT1-1-3*) is dispensable for vegetative growth and sexual reproduction, such as asci production (Ferreira et al., 1998). However, the mutant A^IIRIP^, which contains mutations in both *mat A-2* and *mat A-3* (Glass and Lee, 1992; Ferreira et al., 1996), normally mates as a *mat A* strain, but produces very few asci with ascospores (Ferreira et al., 1998). The *FMR1*, *SMR1* and *SMR2* (*MAT1-1-3*) are required for the development of fertilized female organs in *P*. *anserine*, *SMR2* (*MAT1-1-3*) was not essential for vegetative growth (Debuchy et al., 1993). However, the homothallic *S*. *macrospora SmtA-3* (*MAT1-1-3*) is not essential for fertility and vegetative growth (Klix et al., 2010), and *F*. *graminearum MAT1-1-3* is not essential for perithecium formation and vegetative growth, but is required for ascosporogenesis in self-crosses (Zheng et al., 2015). In our study, we demonstrated that knockout of *MAT1-1-3* caused obvious growth and conidial morphology defects compared to wild type. Additionally, the Δ*mat1-1-3* mutant could not form sclerotia after infecting rice spikelets. Meanwhile, *MAT1-1-3* is required for mating hyphae growth. A recent study showed that the *MAT1-1-3* of *F. graminearum* is not required for virulence (Zheng et al., 2015). However, *MAT1-1-3* was essential for the full pathogenicity of *V. virens*. Fungal sclerotia, which usually form in response to adverse environments like winter, germinate to form hyphae that serve as specific structures to start sexual reproduction under suitable conditions, thereby acting as the primary inoculum in the disease cycle (Yong et al., 2018; Liu et al., 2019). In outcrosses, the Δ*mat1-1-3* mutant was unable to produce sclerotia and could not initiate sexual reproduction. In addition, MAT1-1-3 interacted with MAT1-1-1, which is consistent with the MAT proteins in *Saccharomyces cerevisiae* and *Phaffia rhodozyma* to form complexes to regulate function (Haber, 2012; David-Palma et al., 2016). Thus, the *MAT1-1-3* plays a key role in asexual development, sexual reproduction and pathogenicity of *V. virens*. Fungal fruiting bodies are important structures for fungi development. The fruiting bodies formed by *Cordyceps. spp.* have an important value as edible and medicinal mushrooms (Zheng et al., 2011, 2013). Several studies reported that the mechanisms of regulating fungal fruiting body formation are different. For example, the *MAT1-2* single mating-type strain of *Ophiocordyceps xuefengensis* could form sterile fruiting bodies, with a *MAT1-2* locus that is only *MAT1-2-1* (Zou et al., 2017). The heterothallic *C. militaris* still can produce perithecia and ascospores although the loss of *MAT1-1-3* in the genome in outcrosses (Zheng et al., 2011). The mature fruiting bodies were formed after being crossed with two opposite strains. However, this is the first ascomycete species reported that the *C. militaris* Cm01 stain with only a *MAT1-1* single mating-type idiomorph also can produce stroma (fruiting body) without mature perithecia and ascospores on caterpillar pupae (Zheng et al., 2011). This is consistent with our result that the Δ*mat1-1-3* mutant can produce fruiting body primordium on PSA medium plate, but the fruiting body could not reach maturity and produce ascospores. Altogether, the result indicates that *MAT1-1-3* is required for regulating fruiting bodies development in *V. virens*. Furthermore, a recent study showed that during outcrossing in *C. militaris*, *MAT1-2-1* is required for fruiting body formation, and *MAT1-1-2* mutant could produce fruiting body but with sterile perithecia (Lu et al., 2016). These results suggest that the fruiting body also can be regulated by other mating-type genes, which provides an important basis for us to study the function of other mating-type genes in different fungi.

### *MAT1-1-3* Regulates the Expression of a Subset Genes Involved in MAPK Pathway, Cell Wall Integrity, Epigenetic Modification and Autophagy

Mitogen-activated protein kinase (MAPK) cascades are a conserved signal pathway that has been shown to play a key role in transduction extracellular signals to cellular responses (Gustin et al., 1998; Goldsmith and Dhanasekaran, 2007). Meanwhile, the G proteins perceive the extracellular signal through cell surface receptors, such as G-protein-coupled receptor (GPCR), which are critically involved in the regulation of different MAPK networks (Goldsmith and Dhanasekaran, 2007). MAPK modules regulate many important signaling pathways including cell proliferation, differentiation and apoptosis (Chang and Karin, 2001). Extensive studies showed that MAPK encoding genes are required for sexual reproduction in fungi (Herskowitz, 1995; Banuett, 1998). For example, the MAPK genes are required for fruiting body formation in *Aspergillus* (AN1017) and *Neurospora* (NC02393) (Pöggeler et al., 2006). In yeast, MAPK Slt2p, also called Mpk1p, involved in the regulation of many cellular events including the sexual cycle (Gustin et al., 1998). Moreover, a recent study showed that the blue-light receptor gene white collar-1 (*CmWC-1*) from *C. militaris* mediated the fruiting body development most likely via regulating G protein-coupled receptors (Yang et al., 2016). Strikingly, our RNA-seq and qRT-PCR data showed that the expression levels of *MAF1*, encoding a mitogen-activated protein kinase, *MAPKK* and *GPCR* increased in Δ*mat1-1-3* mutant. Furthermore, *Ga* and *Gb*, encoding G protein alpha and beta subunit respectively, exhibited reduced expression in Δ*mat1-1-3* mutant. Thus, the results indicate that the MAPK signal pathway also participates in the *V. virens* sexual reproduction, although the mechanism of MAPK regulation remains unclear. In addition, *MAT1-1-3* of *V. virens* is also required for regulating the expression of *PRE2* and *PPG1*, encoding pheromone receptor and pheromone precursor, respectively, which are important for fungal sexual reproduction (Pöggeler et al., 2006). Thus, it will contribute to better understand the *V. virens* sexual cycle via studying the function of these genes regulated by *MAT1-1-3*.

The cell wall is essential for growth, signal transduction and participates in morphogenetic and differentiation processes in yeast and fungi (de Medina-Redondo et al., 2008). We found that knockout of *MAT1-1-3* affected the *V. virens* cell wall integrity. Furthermore, *MAT1-1-3* also regulated abiotic stress, such as salt, osmotic- and oxidatives tress. Notably, many genes involved in cell wall integrity and stress response were misregulated in Δ*mat1-1-3* mutant. The *Gel1*, encoding the 1, 3-b-glucanosyltransferase, is required for maintaining the cell wall integrity of *F. graminearum* (Bolouri et al., 2017). Similarly, *Gel2* is required for cell wall morphogenesis and virulence in *Aspergillus fumigatus* (Mouyna et al., 2005). Apparently, these two genes are up-regulated and down-regulated in Δ*mat1-1-3* mutant, respectively. In addition, several genes involved in epigenetic modification can be regulated by MAT1-1-3, such as *DIM-2*, *HMT*, *SET-1* (encoding SET domain-containing protein), *HAT-1* (encoding histone acetyltransferase) and *HDCA* (encoding histone deacetylase A). *SET-2* (a homolog of *HMT* in *V. virens)* is essential for normal growth and development in *Neurospora* (Adhvaryu et al., 2005). Zheng et al. (2011) reported that *C. militaris* fruiting without mating also induced expression of several epigenetic genes. Epigenetic modification plays important roles in silencing and activating different gene clusters, including virulence genes, secondary metabolism and development related genes (Cichewicz, 2010). Thus, the future research on how *MAT1-1-3* regulates these genes expression will expand the sexual development networks. Our RNA-seq and qRT-PCR data also show that the expression of all 7 autophagy-related genes, including *ATG2*, *ATG9*, *ATG12*, *ATG13*, *ATG17*, *ATG22* and *ATG26*, was increased in Δ*mat1-1-3* mutant. The autophagy is an evolutionarily conserved cellular transport pathway involved in many cellular processes (Mizushima et al., 2011). For example, Atg22 plays a vital role in the development and virulence of *F. oxysporum* (Khalid et al., 2019). However, the function of these autophagy-related genes in *V. virens* is still unknown. Taken together, the RNA-seq data indicated that *MAT1-1-3* may regulate developmental processes and virulence by regulating the function of epigenetic modification and autophagy-related genes, which provides a clue for future research on how *MAT1-1-3* regulates the function of these genes.

## Data Availability Statement

All datasets generated for this study are included in the article/Supplementary Material.

## Author Contributions

MYo, JY, and YL designed the study. MYo, XP, MYu, HC, and ZQ performed the experiments. YD, RZ, TS, XY, and ZC helped to analyze the data. MYo, WL, and YL wrote the manuscript. YL contributed to reagents, materials, and analysis tools. All authors contributed to the article and approved the submitted version.

## Conflict of Interest

The authors declare that the research was conducted in the absence of any commercial or financial relationships that could be construed as a potential conflict of interest.

## Footnotes

1 http://grna.ctegd.uga.edu/
