# Supplementary material for: MAT1-1-3, a Mating Type Gene in the Villosiclava virens, Is Required for Fruiting Bodies and Sclerotia Formation, Asexual Development and Pathogenicity
Source: Front Microbiol. 2020 Jun 25;11:1337. doi: 10.3389/fmicb.2020.01337 (PMC7344243; doi:10.3389/fmicb.2020.01337)
Supplement: Supplementary file 1 [file Data_Sheet_1.DOCX]

**Supplementary figures and legends**


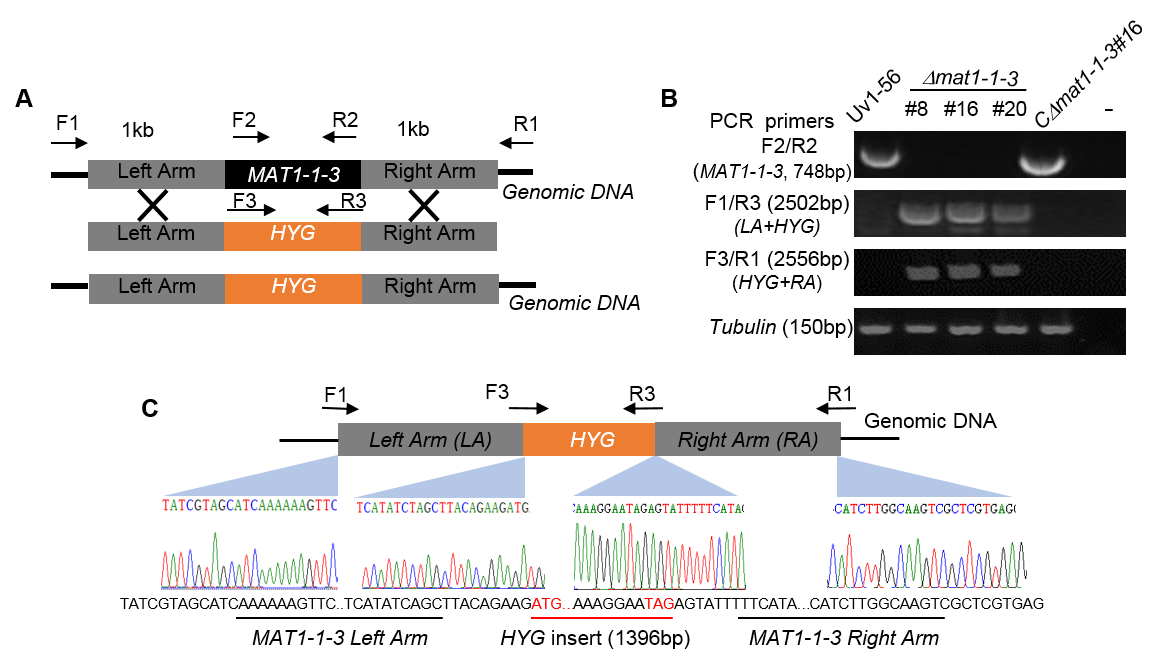


**Figure S1** *MAT1-1-3* was replaced with *HYG* using CRISPR/Cas9 technology. (A) The schematic diagram for *MAT1-1-3* gene replacement using the CRISPR/Cas9 system. The *MAT1-1-3* coding region in wild-type *V. virens* Uv1-56 was replaced with *HYG* (*hygromycin B-resistance*) gene to produce *MAT1-1-3* mutants. The size of the left and right arms was 1 kb. Primer pairs F1/R3, F3/R1 and F2/R2 were used for *MAT1-1-3* mutant screening. (B) Genome DNA PCR analysis demonstrated that *MAT1-1-3* was completely replaced by *HYG* in mutants (#8, #16 and #20). (C) Sanger sequencing traces of junction regions confirmed that *MAT1-1-3* was replaced with *HYG* in mutants. Start and stop codons are indicated in red.


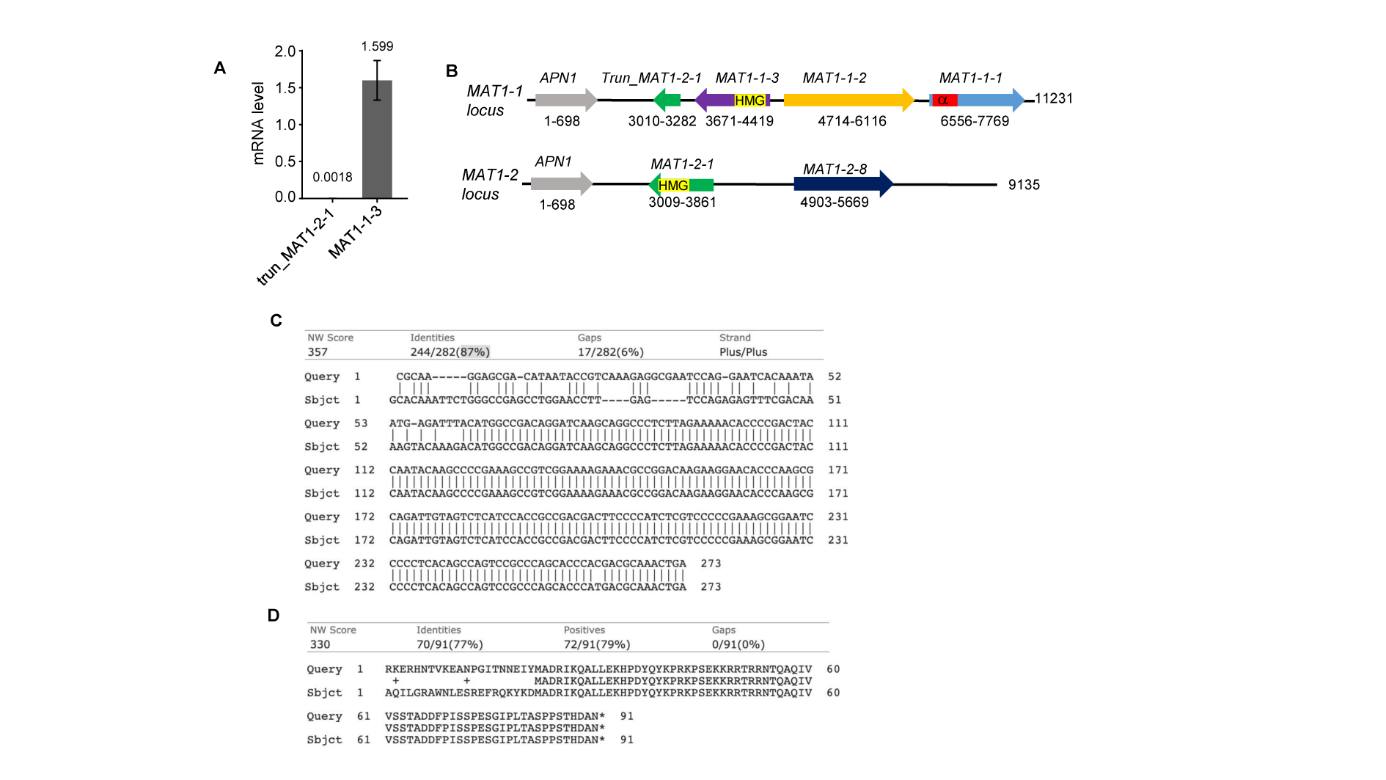


**Figure S2** qRT-PCR analysis of the truncated *MAT1-2-1* (trun_*MAT1-2-1*) and *MAT1-1-3*, schematic diagram of *MAT1-1* and *MAT1-2* loci and multiple sequence alignment of the trun_*MAT1-2-1* and *MAT1-2-1*. (A) The expression of trun_*MAT1-2-1* and *MAT1-1-3* was checked by qRT-PCR. *β*-*tubulin* was used as an internal control and three biological replicates have similar trends. (B) Schematic diagram of *MAT1-1* and *MAT1-2* loci is indicated. The mating type genes were marked with different colour as indicated in figure. (C) Nucleotide sequence alignment of the trun_*MAT1-2-1* and *MAT1-2-1*. (D) Amino acid sequence alignment of the trun_MAT1-2-1 and MAT1-2-1 proteins.
