# Supplementary material for: MAT1-1-3, a Mating Type Gene in the Villosiclava virens, Is Required for Fruiting Bodies and Sclerotia Formation, Asexual Development and Pathogenicity
Source: Front Microbiol. 2020 Jun 25;11:1337. doi: 10.3389/fmicb.2020.01337 (PMC7344243; doi:10.3389/fmicb.2020.01337)
Supplement: Supplementary file 2 [file Table_1.DOCX]

**Table S1** Strains used in this study.

| Strains | Genotype description |
| --- | --- |
| Uv1-56 | Wild type, *MAT1-1* mating-type |
| Uv2-51 | Wild type, *MAT1-2* mating-type |
| *Δmat1-1-3*#8 | *MAT1-1-3* deletion mutant of Uv1-56 |
| *Δmat1-1-3*#16 | *MAT1-1-3* deletion mutant of Uv1-56 |
| *Δmat1-1-3*#20 | *MAT1-1-3* deletion mutant of Uv1-56 |
| *Δmat1-1-1* | *MAT1-1-1* deletion mutant of Uv1-56 |
| *Δmat1-1-2* | *MAT1-1-2* deletion mutant of Uv1-56 |
| MAT1-1-3-GFP transformant | MAT1-1-3-GFP expressing strain |
| C*Δmat1-1-3#16* | *Δmat1-1-3*#16 complemented transformant |
| AH109 | *Yeast* strain |
| AGL1 | *Agrobacterium tumefaciens* strain |
