# Supplementary material for: MAT1-1-3, a Mating Type Gene in the Villosiclava virens, Is Required for Fruiting Bodies and Sclerotia Formation, Asexual Development and Pathogenicity
Source: Front Microbiol. 2020 Jun 25;11:1337. doi: 10.3389/fmicb.2020.01337 (PMC7344243; doi:10.3389/fmicb.2020.01337)
Supplement: Supplementary file 3 [file Table_2.DOCX]

**Table S2** The primers used in this study.

| Primer names | Oligonucleotide sequences (5’-3’) |
| --- | --- |
| *MAT1-1-3*-upF | CTGCCGTTCGACGATTAAAAGTTCCAAACACCAACAGCC |
| *MAT1-1-3*-upR | GCTCCTTCAATATCATCTTCTGTAAGCTAGATATGATCGATCATAG |
| *MAT1-1-3*-doF | CGAGGGCAAAGGAATAGAGTATTTTTCATAGCCTATCTGAAAG |
| *MAT1-1-3*-doR | GGATCTTCCAGAGATTTTGCCAAGATGCTCTGATAAATA |
| F3 | ACAGAAGATGATATTGAAGGAGC |
| R3 | TACTCTATTCCTTTGCCCTCG |
| *MAT1-1-3*-sgRNAF | ACCTAGGTTCGACAACGATCGTATCGG |
| *MAT1-1-3*-sgRNAR | AAACCCGATACGATCGTTGTCGAACCT |
| *MAT1-1-3*-F2 | GAAGGTACGAACTTCTCGAAC |
| *MAT1-1-3*-R2 | TTCAGATAGGCTATGAAAAAATG |
| F1 | AGTGATTCCGCCTTCTCGAT |
| R1 | AAGGCTGGGTTAGGGCAAGCAT |
| C-*MAT-1-1-3*-F | AGTTCTTCTGACCCGGGGATCCTACTCCTGCTCTACGTTTAAT |
| C-*MAT-1-1-3*-R | ACGACGGCCAGTGCCAAGCTTTCATTCACTCTTATCGACATTTTTTCTA |
| *MAT1-1-3*-GFP-F | ACCACAGCAATGGCTGGATCCATGAGACCACGAGTGGAAAT |
| *MAT1-1-3*-GFP-R | CCTTGCTCACCATCCCGGGTTCACTCTTATCGACATT |
| *PPG1*-RT-F | AGTCCGAAGCGAGAAAGACA |
| *PPG1*-RT-R | CTTCTTCAAGCACACGATGC |
| *PRE1*-RT-F | CTGTCCGTCCTCAACATCCT |
| *PRE1*-RT-R | GGCGATGGGTATGTAGCAGT |
| *PRE2*-RT-F | ACAAAAGCAGCCAGAAGTCC |
| *PRE2*-RT-R | GATCGAAATGGTCGAGCTTC |
| *β*-tubulin-F | GGCGTTTACAATGGCACTTC |
| *β*-tubulin-R | CGGAACAGTTGACCAAAAGG |
| *trun-MAT1-2-1*-RT-F | GAGCCTGGAACCTTGAGTCC |
| *trun-MAT1-2-1*-RT-R | TTGGGTGTTCCTTCTTGTCC |
| BD113-F | GGAGGCCGAATTCCCGATGAGACCACGAGTGGAAAT |
| BD113-R | GGTCGACGGATCCCCGGTTCACTCTTATCGACATTTT |
| AD111-F | CAGTGAATTCCACCCGCCCATGGCGACCAGAGCAGAACTTA |
| AD111-R | GTCGTATGGGTACCCACCAAATGTGTAATGGAAATC |
| AD112-F | CAGTGAATTCCACCCGCCCATGGACAGCATTTACCATT |
| AD112-R | GTCGTATGGGTACCCTATGGCCATGAAAGTCGGCAATTG |
